# Effect of ammonium and high light intensity on the accumulation of lipids in *Nannochloropsis oceanica* (CCAP 849/10) and *Phaeodactylum tricornutum* (CCAP 1055/1)

**DOI:** 10.1186/s13068-018-1061-8

**Published:** 2018-03-09

**Authors:** María Huete-Ortega, Katarzyna Okurowska, Rahul Vijay Kapoore, Matthew P. Johnson, D. James Gilmour, Seetharaman Vaidyanathan

**Affiliations:** 1 Department of Chemical and Biological Engineering, Advanced Biomanufacturing Centre, The University of Sheffield, Sir Robert Hadfield Building, Mappin Street, Sheffield, UK; 2 Department of Plant Sciences, University of Cambridge, Downing Street, Cambridge, UK; 3 Department of Molecular Biology and Biotechnology, The University of Sheffield, Firth Court, Western Bank, Sheffield, UK

**Keywords:** Biofuel, Microalgae, High light, Nitrogen limitation, Ammonium, Tungstate, *Phaeodactylum tricornutum*, *Nannochloropsis oceanica*, Nitrate reductase

## Abstract

**Background:**

Microalgae accumulate lipids when exposed to stressful conditions such as nutrient limitation that can be used to generate biofuels. Nitrogen limitation or deprivation is a strategy widely employed to elicit this response. However, this strategy is associated with a reduction in the microalgal growth, leading to overall poor lipid productivities. Here, we investigated the combined effect of a reduced source of nitrogen (ammonium) and super-saturating light intensities on the growth and induction of lipid accumulation in two model but diverse microalgal species, *Phaeodactylum tricornutum* and *Nannochloropsis oceanica*. We hypothesized that the lower energy cost of assimilating ammonium would allow the organisms to use more reductant power for lipid biosynthesis without compromising growth and that this would be further stimulated by the effect of high light (1000 µmol m^−2^ s^−1^) stress. We studied the changes in growth and physiology of both species when grown in culture media that either contained nitrate or ammonium as the nitrogen source, and an additional medium that contained ammonium with tungsten in place of molybdenum and compared this with growth in media without nitrogen. We focused our investigation on the early stages of exposure to the treatments to correspond to events relevant to induction of lipid accumulation in these two species.

**Results:**

At super-saturating light intensities, lipid productivity in *P. tricornutum* increased twofold when grown in ammonium compared to nitrogen free medium that increased further when tungsten was present in the medium in place of molybdenum. Conversely, *N. oceanica* growth and physiology was not compromised by the high light intensities used, and the use of ammonium had a negative effect on the lipid productivity, which was even more marked when tungsten was present.

**Conclusions:**

Whilst the use of ammonium and super-saturating light intensities in *P. tricornutum* was revealed to be a good strategy for increasing lipid biosynthesis, no changes in the lipid productivity of *N. oceanica* were observed, under these conditions. Both results provide relevant direction for the better design of processes to produce biofuels in microalgae by manipulating growth conditions without the need to subject them to genetic engineering manipulation.

## Background

In the current global scenario of climate change and increasing energy demand, it is ever more relevant to conduct research towards development of sustainable and economically efficient ways of energy generation. Microalgae have great potential as a biofuel feedstock and are considered a realistic sustainable alternative to the production of biodiesel from oil crops, waste cooking oil and animal fat. Several reasons justify microalgae use in biofuel generation, such as high growth rates in comparison with terrestrial plants, high biomass and oil yields and ability to grow in non-arable lands and water resources unsuitable for agriculture [1–3]. Microalgae constitute a very diverse group of prokaryotic and eukaryotic photosynthetic microorganisms that can grow rapidly and in very different environments, including those with extreme conditions such as frozen surfaces or waters with very high salinity [4]. The taxonomic diversity of these organisms is one of its most attractive qualities, as it implies the possession of a great variety of metabolic pathways that can potentially be exploited not only for the production of biofuels, but also for products of value in the nutraceutical, pharmaceutical and aquaculture industries [5–7]. However, research conducted in the field has been restricted to a few microalgae model species, with most microalgae diversity still unexplored and with even fewer studies investigating the physiological response of different species under similar culture conditions. Given the taxonomic divergence and the diversity of associated evolutionary lineages and habitats of species broadly classified as microalgae, a cross-species comparison would be immensely beneficial in developing algae biotechnology. Such investigations would enable us to build the conceptual framework for developing strategies towards the effective use of microalgae for biofuel production.

Microalgae produce energy storage molecules that can be used in the biofuel industry, i.e., lipids and carbohydrates, when exposed to stressful conditions such as high salinity or nutrient limitation [4, 8–10]. One of the most widely employed strategies to stimulate microalgae energy storage is nitrogen limitation or deprivation in the growth medium, which has been reported to give the highest triacylglycerol (TAG, the most important lipid biofuel precursor) yields in a wide range of microalgae species [11, 12]. However, this strategy has the associated problem of causing a reduction in the growth of microalgae, leading to a decrease in the biomass yield, and therefore of lipid productivity. To increase microalgae lipid production and at the same time overcome its negative effect on growth, recent research has focused on the development of two strategies. On the one hand, the knowledge of the metabolic pathways involved in energy storage and their regulation has provided the basis for genetic engineering of microalgae into strains with higher lipid productivities [13–16]. On the other hand, to avoid the societal and scientific controversy associated with the commercialization of genetically engineered strains and the possible environmental risks [17–19], studies have also been conducted towards increasing the lipid productivity by manipulating the culture media conditions [9, 10, 20, 21]. For instance, the diatom *Phaeodactylum tricornutum* growing in a reduced form of nitrogen such as ammonium has been shown to produce the same amount of lipids as in the absence of nitrogen without compromising the growth rate [22]. In addition, the use of high light intensities has been described to increase lipid production under nitrogen limited conditions, although this effect seems to be species-specific [23–27]. Adverse conditions that hamper microalgae growth such as nitrogen deprivation cause an energy imbalance as a consequence of the accumulation of photosynthetic reductant power, which can ultimately lead to photo-oxidative damage of photosystems I (PSI) and II (PSII) [15, 28, 29]. To avoid this, TAG biosynthesis is stimulated to act as an alternative sink for the excess reductant power [2, 30]. It is hypothesized that the stress associated with increasing light intensity under conditions of nitrogen deprivation would add a higher pressure on an already damaged photosynthetic pathway, leading to an increase in the energy imbalance, which in turn would stimulate an even higher TAG production [26, 31–33].

The genus *Nannochloropsis* is considered one of the most promising microalgae groups for the production of biofuels, because of its ability to accumulate lipids up to 65–70% of its dry weight [7, 34, 35]. This is higher than those obtained by other species, such as the diatom *P. tricornutum* [36]. It is also an important source of the omega-3 long-chain polyunsaturated fatty acid eicosapentaenoic acid, which is very valuable in the aquaculture industry [37–39]. Microorganisms of this genus belong to the Eustigmatophyceae, which is phylogenetically very distinct from green algae or diatoms and instead closer to groups such as the Chrysophyceae and Xanthophyceae within the phylum Ochrophyta [40]. They tend to live in coastal ecosystems and have small size (approx. 3 µm). In addition, species of this group, such as *Nannochloropsis gaditana* or *N. oceanica*, are emerging model organisms, due to their high growth rates and the development of tools for their genetic manipulation [13, 41]. This group also has distinct characteristics in its photosynthetic pathway. For instance, it has a large chloroplast encircled by four membranes whose pigment composition is composed only of chlorophyll *a* and no chlorophyll *b* or *d*, with violaxanthin and vaucheraxanthin as the predominant carotenoids involved in the xanthophyll cycle [42, 43], and where the ratio of PSII to PSI is 1:1 contrasting with the 2:1 and 1:2-3 ratios of diatoms and cyanobacteria, respectively [28, 44].

In the present study, we aimed to investigate the combined effect of ammonium, a reduced source of nitrogen, and high light intensities in the lipid accumulation of the microalga *N. oceanica*. The results obtained were compared with the response by the diatom *P. tricornutum*, which possesses a very active xanthophyll cycle involving the carotenoids diadinoxanthin and diatoxanthin, a different PSII:PSI ratio of 2:1 [28], and for which its growth in ammonium has been shown to produce lipid yields similar to nitrogen depletion in the medium without compromising growth, at lower sufficient light intensities [22]. In addition, we examined different photosynthetic properties to assess how the energy imbalance generated translated into lipid accumulation. The two chosen species represent taxonomically diverse but closely grouped genera with relative genetic similarities, compared to the more commonly examined green algae and plants. Besides, the mechanism of NPQ in *Nannochloropsis* sp. has been shown to resemble that of diatoms [45]. To the best of our knowledge, this is the first attempt at a species comparison of the physiological changes experienced by these two microalgae under the combined effect of ammonium and high light. The information obtained from this comparative study would be of high value for the microalgal biofuel industry, because it would allow the design of more effective industrial exploitations in which the lipid productivity of these two organisms would be maximized by manipulating their culture conditions without the need for genetic engineering, and would also enable devising strategies for biofuel production from other microalgae species.

## Results

### Changes in the growth curve of *N. oceanica* and *P. tricornutum*

**Fig. 1:**
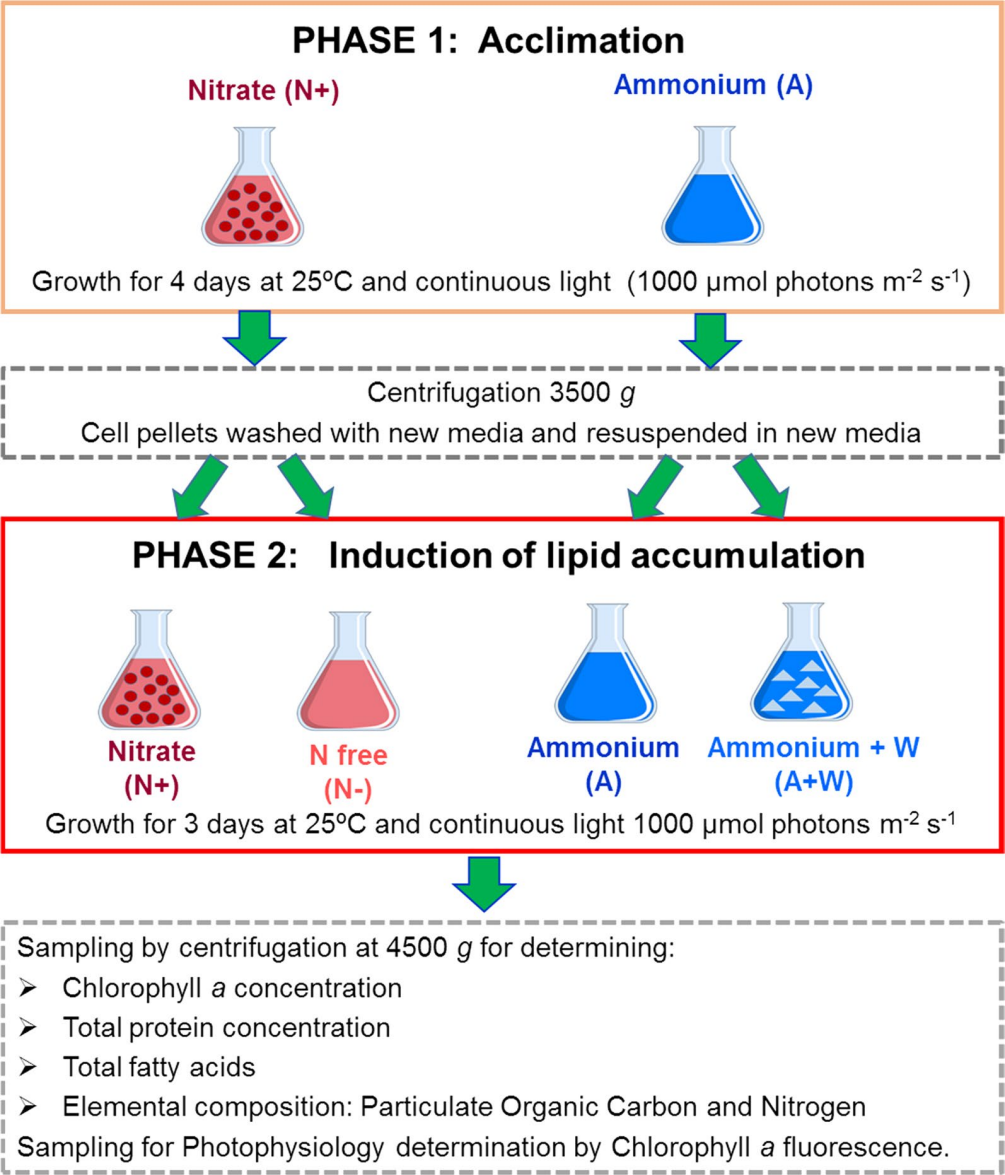
Experimental flow diagram. Each of the species was cultivated in two phases. In phase I, the cells were acclimatised to the nitrogen source and high light intensities, before transferring actively growing cells to the respective experimental medium, as indicated, in phase II. This was followed by sampling for analysis of the parameters as indicated. All the cultivations in both the phases were carried out in triplicates

**Fig. 2:**
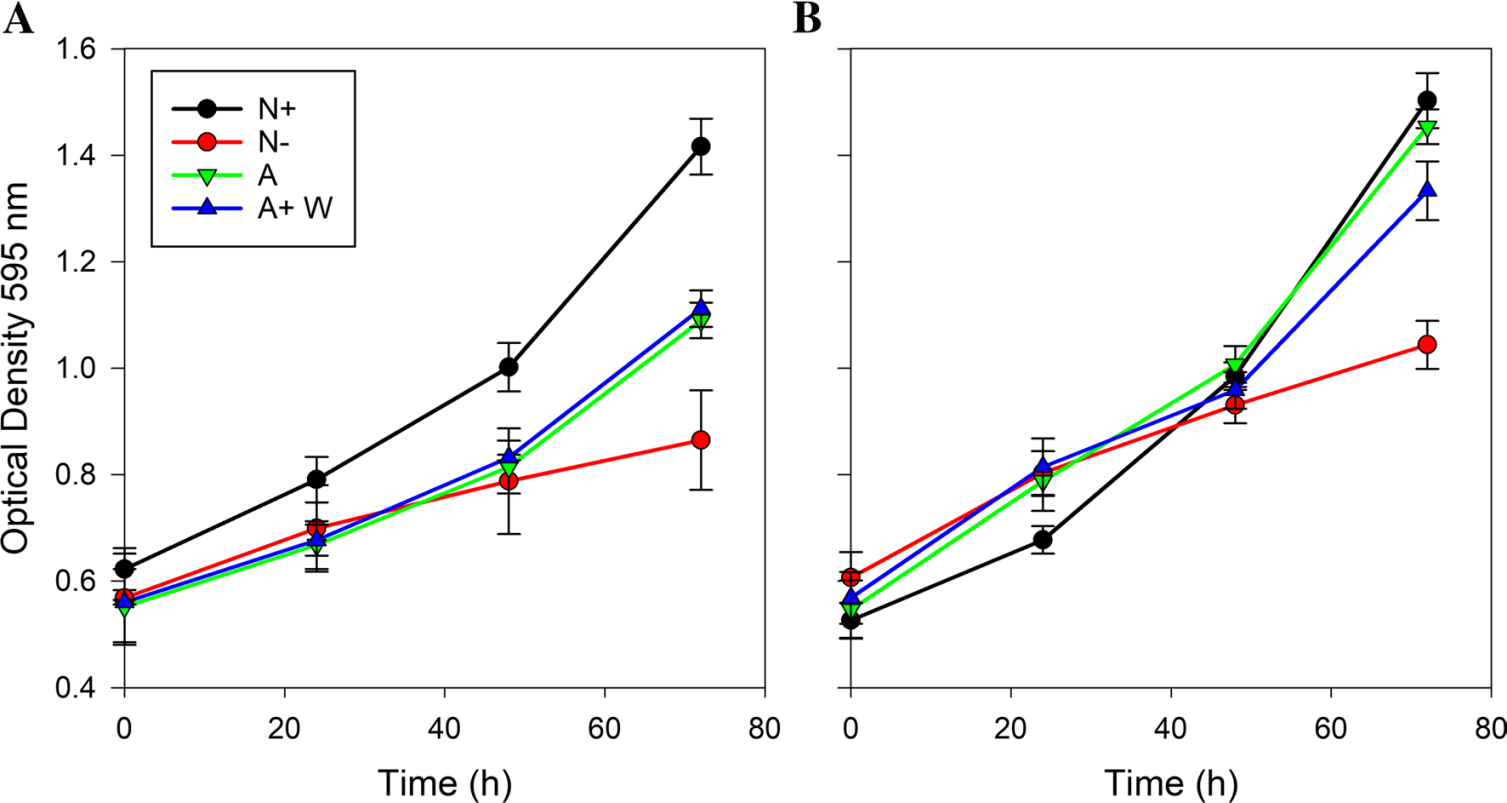
Growth measured by optical density at 595 nm. N+ (black circle), N− (red circle), A (green inverted triangle) and A+W (blue triangle) conditions of **A** *P. tricornutum* and **B** *N. oceanica*

Both the organisms were first grown in high light intensities in f/2 medium containing either nitrate or ammonium as the nitrogen source, in a first phase to acclimatise them in the respective nitrogen medium (Fig. 1). Cells in the active growth phase (optical density (OD_595 nm_) of ~ 0.6) were then washed and transferred to fresh media [nitrate to nitrate replete (N+) and nitrogen free (N−) and ammonium to ammonium (A) and ammonium with tungstate (A+W)], and allowed to grow in a second phase for 3 days (Fig. 1). Optical density was used to measure growth, as it enabled usage of a small sample volume for measurements and it showed good correlations to cell abundance and dry cell weight measurements, for the two species established with larger sample volumes. The cultures were harvested after 3 days of growth in phase 2 to ensure that physiological changes relevant to induction of lipid accumulation were monitored and minimise compounding effects such as influence of cell shading from photoinhibition at higher cell densities and nutrient remineralisation in the medium. Besides, the standard differential physiological response expected in the N+ and N− conditions was evident in the 3 day cultures. *N. oceanica* reached higher optical densities and cell abundances after 3 days in the second phase compared to *P. tricornutum*, when grown in the N+ and N− treatments (Fig. 2 and Table 1), but both species showed a nearly twofold decrease in growth rate in N− compared to the N+ treatment (ANOVA analysis with Bonferroni post hoc test, *p* value < 0.05). However, differences were observed in cell abundances and growth rates achieved by the two species under the other treatments tested. As can be seen in Table 1, *P. tricornutum* presented similar growth rates and cell abundances in the A and A+W treatments, which were slightly lower than in N+ and not significantly different to those in the N− treatment. In contrast, the cell abundances of *N. oceanica* in the A and A+W treatments were quite similar to that in the N+ treatment, although the biological variability of replicates failed to point out significant differences between their growth rates and those of the N+ and N− treatments. In addition, both species also showed differences in the evolution of the optical density over time (Fig. 2), with values very similar in *P. tricornutum* up to 48 h for the N−, A and A+W treatments and for these three treatments and the N+ in *N. oceanica.* The N− treatments at 72 h showed deviations from the other treatments for both the species, indicating the effects of nitrogen limitation.

**Table 1:** Cellular characteristics of *N. oceanica* and *Phaeodactylum tricornutum*

Species	*P. tricornutum*				*N. oceanica*			
Treatment	N+	N−	A	A+W	N+	N−	A	A+W
Cell abundance (×10^6^ cells mL^−1^)	20.4	6.25	11	10	48.1	24.5	5.64	36.1
µmax (day^−1^)	0.35 (± 0.02)	0.21^(a)^ (± 0.02)	0.29 (± 0.03)	0.29 (± 0.02)	0.42 (± 0.01)	0.29^(a)^ (± 0.03)	0.38^(b)^ (± 0.03)	0.36^(b)^ (± 0.02)
POC (µg mg^−1^)	328.4 (± 21.7)	310.6 (± 24.2)	345.5 (± 32.1)	288.9 (± 7.9)	503.2 (± 7.9)	590.1^(a)^ (± 8.8)	576.2^(a)^ (± 1.0)	534.8 (± 18.6)
PON (µg mg^−1^)	32.8 (± 1.6)	20.3^(a)^ (± 0.9)	28.5^(b)^ (± 1.0)	26.5^(a, b)^ (± 1.0)	48.4 (± 0.6)	23.0^(a)^ (± 1.0)	43.1^(b)^ (± 0.6)	45.2^(b)^ (± 2.2)

Maximum growth rate (µmax), cell abundance, particulate organic carbon (POC) and particulate organic nitrogen (PON) are compared for the two species in the four conditions tested [nitrate (N+), nitrogen free (N−), ammonium (A) and ammonium with tungstate (A+W) conditions]. Mean (± standard error) values are given. Analysis of the variance followed by Bonferroni post hoc test (*p* value < 0.05) was carried out to estimate the significance of the differences between treatments, being (a) significant difference when comparing to the N+ treatment, (b) significant difference when comparing to the N− treatment

### Biochemical composition and carbon and nitrogen assimilation of *N. oceanica* and *P. tricornutum*

**Fig. 3:**
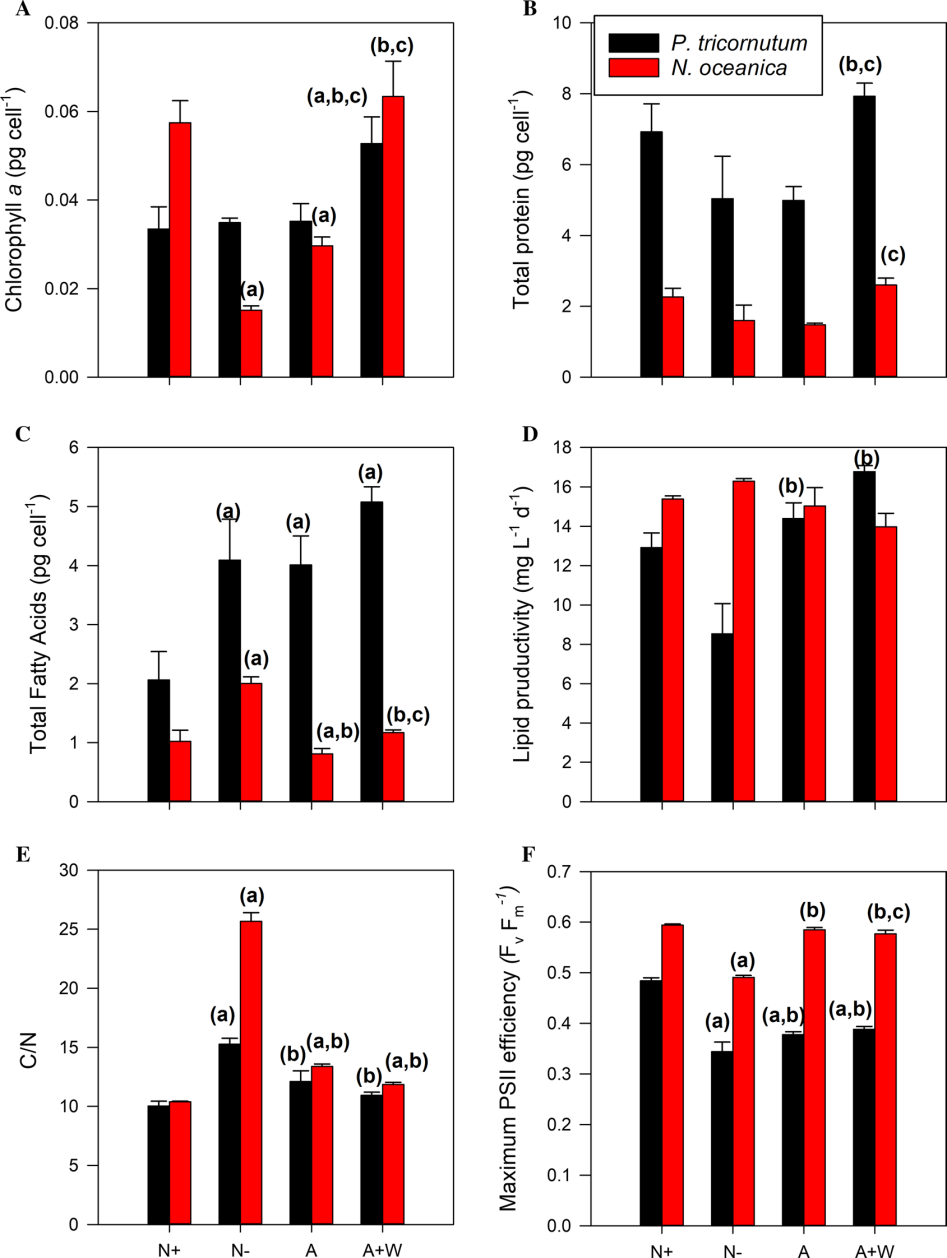
Biochemical composition and maximum photosynthetic PSII efficiency estimated at the end of the experiment (day 3). *Phaeodactylum tricornutum* (black bars) and *N. oceanica* (red bars) comparisons for **A** cell-specific chlorophyll *a* concentration (pg cell^−1^), **B** cell-specific total protein concentration (pg cell^−1^), **C** cell-specific total fatty acids (pg cell^−1^), **D** lipid productivity (mg L^−1^ day^−1^), **E** Carbon to nitrogen ratio and **F** maximum photosynthetic PSII efficiency (*F*_v_/*F*_m_) for the two species in the four conditions [nitrate (N+), nitrogen free (N−), ammonium (A) and ammonium with tungstate (A+W) conditions]. Mean (± standard error) values are given. Analysis of the variance followed by Bonferroni post hoc test (*p* value < 0.05) was carried out to estimate the significance of the differences between treatments, being (a) significant difference when compared with the N+ treatment, (b) significant difference when compared with the N− treatment and (c) significant difference when compared with the A treatment

In Fig. 3, we can observe important differences in the biochemical composition after 3 days of growth between the two species. Higher chlorophyll *a* (chl a) concentrations were achieved in general in *N. oceanica*, with the significantly higher values observed in the N+ (0.06 ± 0.01 pg cell^−1^) and A+W treatments (0.06 ± 0.01 pg cell^−1^), and the lowest (0.02 ± 0.003 pg cell^−1^) in the N− treatment (Fig. 3A), the latter being a threefold change when compared to the N+ treatment. *P. tricornutum* chl a concentrations were very similar between the N+, N− and A treatments (0.035 ± 0.01 pg cell^−1^). However, a statistically significant increase of chl a in the A+W treatment (0.053 ± 0.01 pg cell^−1^) was also observed.

*N. oceanica* presented lower protein contents than *P. tricornutum* and the protein content in N− and A treatments seemed to be lower than the N+ treatment in both species (Fig. 3B), although these differences were not statistically significant. Interestingly, in both species, the A+W treatments showed significantly higher concentrations of total protein than the A treatment (almost 1.5 times more), with values even higher than the N+ replete treatment.

*P. tricornutum* achieved higher cellular yields of total fatty acids than *N. oceanica* (Fig. 3C), with values twofold higher in N−, A and A+W treatments, compared to the N+ treatment, at the 3 day harvest stage. In contrast, a different behaviour was observed for *N. oceanica* which showed higher cellular yields only in the N− treatment (2.0 ± 0.1 pg cell^−1^) that was twofold higher when compared to the other treatments, and with the lowest cellular yields determined in the A treatment. However, when lipid productivity was calculated, *N. oceanica* showed higher productivities than *P. tricornutum* in all the treatments with the exception of the A+W (Fig. 3D) in which the diatom achieved highest productivities (17 mg L^−1^ day^−1^), higher than in the N− treatment.

Finally, *N. oceanica* showed in general higher contents of particulate organic carbon (POC) and particulate organic nitrogen (PON) than *P. tricornutum*, indicating that this species was better at assimilating carbon and nitrogen than the diatom, under the tested conditions (Table 1). As expected, the N− treatment seemed to significantly affect nitrogen assimilation in both species, although the reduction in PON was higher in *N. oceanica.* The A and A+W treatments also seemed to cause a decrease in the nitrogen assimilation when compared to the N+ treatment, although this was not statistically significant. The N− and A treatments showed higher carbon content in *N. oceanica*, while POC contents in *P. tricornutum* were very similar across all the treatments. As a result, the POC to PON ratio (*C*/*N* ratio) (Fig. 3E), a proxy for the degree of intracellular limitation, was approx. 10 in the N+ replete treatment in both species and increased by 2.5- and 1.5-fold in the N− treatment in *N. oceanica* and *P. tricornutum*, respectively. The changes in the *C*/*N* ratio observed in the A and A+W treatments, when compared to the N+ treatment, were also similar for the two species, showing a modest and statistically insignificant increase in *P. tricornutum*, but a statistically significant increase in *N. oceanica*.

### Photophysiological changes of *N. oceanica* and *P. tricornutum*

The maximum efficiency of PSII in dark-adapted state, *F*_v_/*F*_m_, a sensitive indicator of the maximum quantum efficiency of the PSII, was always slightly higher in *N. oceanica* than in *P. tricornutum* (Fig. 3F). In both species, the N− treatment caused a significant decrease in the photosynthetic efficiency when compared to the rest of the treatments, and in *P. tricornutum*, this decrease was also observed in the A and A+W treatments when compared to the N+ treatment. In contrast, *N. oceanica* photosynthetic efficiency in the A treatment was not significantly different to N+, but slightly higher than in the A+W treatment.

**Fig. 4:**
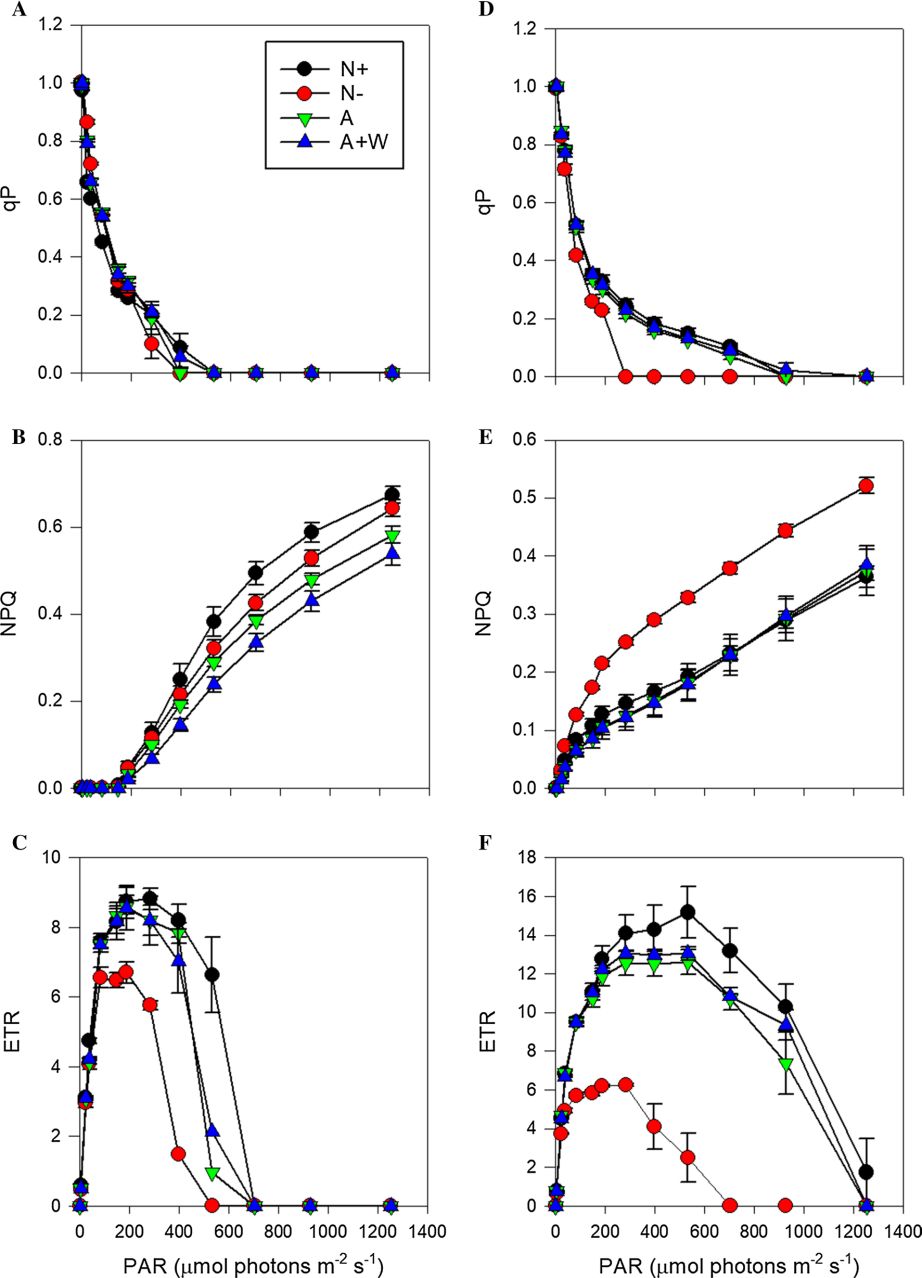
Rapid light response curves estimated by chlorophyll *a* fluorescence. N+ (black circle), N− (red circle), A (green inverted triangle) and A+W (blue triangle) conditions of **A**–**C** *Phaeodactylum tricornutum* and **D**–**F** *N. oceanica*. Mean values ± standard error. qP, photochemical quenching; NPQ, non-photochemical quenching; ETR, electron transport rate. Data not normalized

Differences between the two species in the response of the photophysiological parameters under increasing light intensities were also observed at the end of the experiment (Fig. 4). For instance, qP, a proxy of the proportion of PSII reaction centres that are open, decreased rapidly with increasing actinic light intensities in all the treatments of *P. tricornutum* (Fig. 4a), indicating that in this species PSII activity was saturated at light intensities higher than 400–500 µmol photons m^−2^ s^−1^, independent of the source of nitrogen. In contrast in *N. oce*a*nic*a, PSII reaction centers remained in a more open state in all treatments, except in the N− treatment (Fig. 4d). This suggests that *N. oceanica* experienced a relatively lower excitation pressure than *P. tricornutum*. Differences between both species were also observed in the NPQ and ETR evolution. NPQ and ETR are considered to give a measure of the energy dissipation by heat and that of gross photosynthesis, respectively. The NPQ response in *P. tricornutum* did not start until aprox. 200 µmol photons m^−2^ s^−1^ irradiance (Fig. 4b) and showed more differences between the treatments. The highest NPQ was observed in the N+ treatment and the lowest in the A+W one. In contrast in *N. oceanica*, NPQ increased steadily from the first actinic light intensity in N+, A and A+W treatments (Fig. 4e), and it was much higher and steeper in its response in the N− treatment. In general, NPQ values were higher in *P. tricornutum* compared to *N. oceanica*, for all the treatments. Photosynthesis rates, indicated by the ETR evolution, were much higher in *N. oceanica* (Fig. 4d–f) and both species coincided in the light intensity at which the saturating rates were achieved (optimum light, Table 2). Both species also showed their highest and lowest *P*_max_ in the N+ and N− treatments, respectively (Table 2), although *N. oceanica* was able to conduct photosynthesis at higher light intensities than *P. tricornutum.*

**Table 2:** Light saturation curve parameters fitted to the rapid light curve and qP recovery (ΔqP) after rapid light curve

Species	*P. tricornutum*				*N. oceanica*			
Treatment	N+	N−	A	A+W	N+	N−	A	A+W
Maximum photosynthesis (*P*_max_)	9.81 (± 0.64)	7.36 (± 0.21)	9.20 (± 0.21)	9.20 (± 0.64)	16.18 (± 0.92)	7.48 (± 0.12)	14.72 (± 0.64)	15.08 (± 0.21)
Optimum light (µmol m^−2^ s^−1^)	213 (± 2)	125 (± 6)	167 (± 3)	169 (± 16)	342 (± 8)	152 (± 2)	304 (± 8)	316 (± 6)
Maximum photosynthetic efficiency (*α*)	0.13 (± 0.01)	0.16 (± 0.01)	0.15 (± 0.01)	0.15 (± 0.01)	0.13 (± 0.01)	0.13 (± 0.01)	0.13 (± 0.01)	0.13 (± 0.00)
*r* coefficient	0.94	0.97	0.96	0.97	0.95	0.94	0.95	0.95
ΔqP	0.10	0.03	0.08	0.03	0.0006	0.006	0.009	0.005

Mean (± standard error) are listed

## Discussion

### High light intensities and ammonium with tungstate increase lipid productivity of *P. tricornutum*

In this study, a super-saturating light intensity (1000 µmol photons m^−2^ s^−1^), over threefold higher than that employed in a similar set up by Frada et al. [22], was used. This was done with the idea of testing the hypothesis that high light intensities might further stimulate lipid biosynthesis by increasing the energetic imbalance caused by changes in the ATP and NADPH levels inside the cell. It was reasoned that this would be caused by increasing the excitation pressure on the PSII (that is a higher degree of PSII reduction), especially under nitrogen limitation conditions, which in turn would increase the reductant power (NADPH). The increased reductant power would then lead to the activation of energy dissipation mechanisms such as NPQ (via increases in cyclic electron flow) or lipid biosynthesis [26, 31, 32, 46].

#### Super-saturating light intensities under nitrogen starvation conditions inhibited growth in *P. tricornutum* but lipid productivity remained unchanged

The absence of nitrogen affected the growth of *P. tricornutum* significantly, with a reduction in the maximum growth rates that were nearly halved compared to the ones achieved in the presence of nitrate as a nitrogen source. This is similar to earlier observations made at such super-saturating light intensities [46]. The maximum growth rates achieved in the absence of nitrogen were remarkably similar between reports, whether at sufficient light intensities [15, 22, 47], or at higher super-saturating light intensities ([46], and this report, Table 3). However, the maximum growth rates observed in our study for nitrogen replete conditions (independent of the use of nitrate or ammonium as the nitrogen source) were reduced to nearly a third of that at sufficient light intensities [22]. This indicated that the super-saturating light intensities used are not optimal from a growth perspective, along expected lines.

**Table 3:** Cellular characteristics of *P. tricornutum* reported in (I) Frada et al. [22] (300 µmol photons m^−2^ s^−1^) and (II) Wagner et al. [46] (1000 µmol photons m^−2^ s^−1^)

	(I)				(II)	
	N+	N−	A	A+W	N+	N−
Cell abundance (×10^6^ cells mL^−1^)	2.3	0.8	3	0.8	n.d	n.d
µmax (day^−1^)	0.97 (± 0.10)	0.21 (± 0.04)	0.87 (± 0.05)	1.02 (± 0.03)	0.35	0.18
Chl a (×10^−2^ pg cell^−1^)	20.82 (± 1.84)	4.08 (± 0.30)	45.92 (± 6.9)	23.21 (± 10.5)	n.d	n.d
Fa (pg cell^−1^)	2.39 (± 0.10)	4.21 (± 0.33)	4.33 (± 0.60)	3.35 (± 0.17)	n.d	n.d
Fa productivity (mg L^−1^ day^−1^)	1.83	1.12	4.33	3.13	n.d	n.d
*C*/*N*	7.11	14.64	6.62	6.90	n.d	n.d
*F*_v_/*F*_m_	0.61 (± 0.10)	0.26 (± 0.04)	0.58 (± 0.10)	0.58 (± 0.06)	n.d	n.d

Maximum growth rate (µmax), cell abundance, chlorophyll *a* (chl a), total fatty acids (Fa), total fatty acids productivity (Fa productivity), carbon to nitrogen ratio (*C*/*N*), and photosynthetic maximum efficiency of PSII (*F*_v_/*F*_m_) are compared for *P. tricornutum* in the four conditions (nitrate (N+), nitrogen free (N−), ammonium (A) and ammonium with tungstate (A+W) conditions), as reported in [22] and [46]. Mean (± standard error) values are given. n.d., means no data available

This could also be inferred from the chl a content and cellular *C*/*N* ratio in nitrogen deplete compared to replete conditions. Previous studies have reported a decrease in the chl a concentrations under nitrogen scarcity in many microalgae species [29, 48, 49], and in our study, the chl a content in the N− treatment was similar to that described previously. This appears to be irrespective of the light intensity used, whether it is sub-saturating [50], sufficient [22], or super-saturating light intensities [46]. This reduction in chl a concentrations would be explained by the need for scavenging nitrogen inside the cells, redirecting it to other more important metabolic pathways, as pigments have high requisites for nitrogen [48, 51–53]. In nitrogen replete conditions, the chl a content was much lower under super-saturating light intensities (this report) compared to the ones reported at sufficient light intensities [22] (Table 3). This would suggest a reduced requirement of chl a in excess light, as has been described before for other species in which the acclimation to high light intensities involved the reduction of light harvesting proteins to maintain the photosynthetic carbon assimilation [54, 55]. The cellular *C*/*N* ratio was also observed to be higher in the N+ condition under super-saturating light intensities compared to the earlier study in sufficient light intensities [22] (Table 3), whilst they were comparable for the two light intensities in nitrogen deplete conditions.

The lower growth rates for all treatments, reduced chl a levels, increased *C*/*N,* and the photophysiological measurements conducted at the end of the experiment would point towards cellular photoinhibition. For instance, the number of open photosynthetic reaction centres, qP, and the gross photosynthesis rates, measured as ETR, decreased to zero levels at 533 µmol photons m^−2^ s^−1^ for all treatments, and the maximum efficiency of the PSII values (*F*_v_/*F*_m_) measured for all of them were also lower than those reported previously for the same species [15, 22] (Fig. 4 and Table 3). In addition, the super-saturating light used in this study might have also damaged the PSII, as qP was not able to recover its original values after the RLC measurements (ΔqP were > 0.01 in all the cases) and the NPQ evolution was quite high under high actinic light intensities, indicating a very active xanthophyll cycle that was even significant in the N+ treatment.

Despite the effect of super-saturating light intensities on cellular growth, the cellular lipid content (measured here as total fatty acids (Fa)) showed a similar response in these conditions to those at sufficient light intensities, when comparing N+ and N− treatments [22] (Fig. 3C and Table 3). The values were comparable and a nearly twofold increase in cellular Fa content was observed, although it was marginally higher at the higher light intensities. However, the decreased growth rates in the nitrogen starved conditions, and subsequently decreased biomass yield reported here as cell abundance, resulted in a reduction in the lipid productivities, which showed a 60 and 66% reduction in N− treatment compared to N+, under sufficient and super-saturated light intensities, respectively; the increased light intensity contributing to a greater reduction. The lipid productivities achieved in our investigation were an order of magnitude higher than that reported by Frada et al. [22], primarily as a result of the cell abundances reached in our investigation, which were an order of magnitude higher.

#### The use of ammonium as a source of nitrogen in *P. tricornutum* maximized lipid productivity in the presence of super-saturating light intensities

A previous study showed the use of a reduced form of nitrogen (ammonium) to be effective in increasing cellular lipid levels without compromising growth in *P. tricornutum* [22]. We attempted to extend this finding at super-saturating light intensities, reasoning that the higher light intensity used would accentuate the effect of the ammonium leading to higher lipid levels and productivities. Whilst the growth rates under sufficient light intensities were comparable for the A and A+W treatments with the N+ condition [22] (Table 3), these were lower in A and A+W treatments compared to the N+ condition under super-saturated light conditions (Table 1). In addition, the excess light used in this investigation resulted in markedly lower cellular chl a values and higher *C*/*N* ratios in A and A+W (Fig. 3A, F), compared to a previous report for sufficient light intensities [22] (Table 3). Finally, the maximum efficiency of PSII (*F*_v_/*F*_m_) was lower for the A and A+W conditions compared to the N+ treatment under super-saturating light intensities (Fig. 3F), whilst no differences between the three treatments were observed under sufficient light (Table 3). As described above for the N+ and N− treatments of this study, all these results were likely due to the photoinhibition of the photosynthetic pathway by the excess light, a situation in which the cellular machinery would be cutting down resources for photosynthesis and re-routing carbon and reducing equivalents towards lipid biosynthesis. However, despite the photoinhibitory effect of the light intensities used in this study and contrasting with that observed for sufficient light [22], chl a content was not reduced in the A+W treatment and it was even significantly higher than in the rest of the treatments (Fig. 3A). In addition, W also appeared to somehow influence the regulation of protein synthesis positively, as indicated by the increase in cellular protein concentrations in the A+W treatment that contrasted with the absence of significant changes in the A treatment (Fig. 3B). W tends to replace Mo in the enzyme active sites, and, although traditionally has been used as a biochemical inhibitor of nitrate reductase (NR) [56–59], it is not clear if the activity of other enzymes such as the xanthine dehydrogenase or the aldehyde oxidase are not affected [60]. As far as we know, no effect of W in the chl a biosynthesis or the protein synthesis pathways have been reported, but our result would indicate that some enzymes involved in their regulation might be affected and that, somehow, the W inhibitory effect might interfere in the cellular acclamatory mechanisms to light stress. However, further studies addressing this hypothesis should be conducted to confirm it.

Despite the reduced growth rates obtained under super-saturated light intensities in our study, a significant increase in cellular lipid content and lipid productivities were observed in both ammonium treatments when compared to the N+ treatment (Fig. 3C, D), which coincides with that described in previous studies for sufficient light intensities [22] (Table 3). However, our study contrasted with that of Frada et al. [22] in the fact that lipid content and productivities showed the maximum values in the A+W treatment. Nitrate reduction acts as a sink of photosynthetically generated reductant power inside the cells [2, 30, 46] and, under high light, NR and nitrite reductase are up- or down-regulated depending on the nitrogen source. For instance, in diatoms under high light and in the presence of nitrate, both enzymes are up-regulated, acting as a mechanism to dissipate the excess of photosynthetic energy that could lead to photo-oxidative damage [61–63], while the opposite trend is observed in the presence of ammonium [63–65]. In the latter case, lipid biosynthesis would act as an alternative mechanism for the sink of the excess of photosynthetic energy, which would explain the increase in the lipid production observed in the ammonium treatments, as has been noted earlier [22]. However, the degree of stimulation in lipid productivity observed for those treatments was lower in super-saturating light intensities compared to sufficient light conditions due to the photoinhibition effect on growth, suggesting that an intermediate high light level would elicit a more effective productive response.

In conclusion, our results suggest that it might be possible to combine the use of higher than saturated light and a reduced form of nitrogen such as ammonium instead of nitrate to improve lipid productivity in *P. tricornutum*, without compromising growth. In addition, elucidating the role of W in the presence of ammonium might help in developing strategies that would result in increasing lipid productivities using microalgae.

### High light intensities and ammonium as a source of nitrogen did not stimulate lipid biosynthesis in *N. oceanica*

*N. oceanica* showed very similar responses to those of *P. tricornutum* in the N− treatment. As described in previous studies of this genus [44, 66], the maximum growth rate in the N− treatment nearly halved when compared to the N+ treatment. This observation also coincided with (a) the expected significant decrease of the chl a content and the maximum quantum efficiency of PSII, (b) an increase in the NPQ response under increasing irradiances, and (c) a *C*/*N* ratio 2.5-times higher, as reported previously for similar studies [44, 48, 67]. However, in contrast with *P. tricornutum*, neither N+, A nor A+W treatments seemed to be photoinhibited by the high light intensities used and they were able to reach similar cell abundances at the end of the experiment (3 days). For instance, the maximum quantum yield of PSII was high and very similar between the treatments; a high proportion of the reaction centres remained open until quite high light intensities, closing completely at approx. 900 µmol photons m^−2^ s^−1^; and the gross photosynthesis rates (ETR) were almost twofold higher than those achieved by the N− treatment and did not decrease until higher light intensities (Fig. 4D–F). In addition, the NPQ responses to irradiance were very similar between these treatments and importantly lower than the N− treatment, indicating that cells were not under light stress [26, 44, 55]. All these results would concur with previous reports of the ability of *Nannochloropsis* to acclimate to very high light intensities [26].

The N− treatment also stimulated a twofold increase in the cellular lipid levels when compared to the N+ treatment, although this stimulation was not as high as the almost fourfold higher lipid content reported for this genus under nitrogen deprivation and similar time scales, but under much lower light intensities (100 µmol m^−2^ s^−1^) [44, 68]. In addition, lipid productivities were not significantly different between all the treatments, although in the N− treatment, it was slightly higher than the rest. Therefore, no further stimulatory effect of lipid biosynthesis due to the energetic imbalance caused by the high light stress was observed, which would agree with previous studies done in *N. gaditana* [26]. In addition, although photophysiologically, *N. oceanica* seemed to be less affected by the high light intensities, in contrast to *P. tricornutum*, the use of a reduced form of nitrogen in the presence of high light did not stimulate lipid biosynthesis. In fact, a negative effect on this energetic storage pathway by the use of ammonium was observed. For instance, lipid biosynthesis did not increase in the A+W treatment when compared to the N+ treatment, and it was also significantly lower in the *N. oceanica* cells grown in the A treatment. This also coincided with changes in the biochemical composition, which involved a significant decrease in the chl a content, lower concentrations of total proteins and a significant increase in the *C*/*N* ratios and carbon content when compared to the N+ treatment. Despite the known ability of *N. oceanica* to grow in ammonium [69], all these results would indicate that the available reductant power not used in the nitrate assimilation would be diverted to pathways other than lipid biosynthesis, causing some kind of resource re-allocation inside the cells that occurred without affecting the growth rate and the biomass accumulation. An investigation at a higher light intensity and on the changes at the metabolic level associated with growth in the A and A+W treatments could perhaps give a better insight into the type of response shown by *N. oceanica*. This remains to be investigated.

### Inactivation of nitrate reductase in *P. tricornutum* and *N. oceanica* might reveal species-specific differences in the linkage between photosynthesis and lipid biosynthesis pathways

In the present study, W instead of Mo was used to chemically inactivate the nitrate reductase enzyme, as in the absence of Mo, W tends to occupy the active site of this enzyme [56–59]. Therefore, the use of W instead of Mo in the presence of nitrate would render enzymatically similar results as the N− treatment, while in the presence of ammonium (A+W treatment), this would imply the chemical rescue of the cells from nitrogen starvation, leading to similar results to those obtained for the A treatment [22]. However, very different responses of those expected were observed at the high light intensities tested, which also depended on the species. In *P. tricornutum* the A+W treatment showed higher lipid biosynthesis than the A treatment, despite the fact that growth rates and cell abundances were similar in these two treatments. These observations would coincide with previous studies [22], which suggested that the A+W treatment would be an exaggerated version of the A treatment. It has been reported that the creation of knock-down mutants in *P. tricornutum* for the NR increases TAGs production with a reduction of the growth rate by only 30% [15, 70]. The explanation for this would be that the inactivation of the NR, whether by the ammonium presence or by the use of W, would cause an increase in the glutamate/glutamine (GLU/GLN) ratio and a change in the redox state of the plastoquinone pool that would lead to a signalling cascade resulting in redirecting photosynthetically fixed carbon into lipids [15, 47, 70]. In contrast, in *N. oceanica* the opposite effect was observed, with lipid contents in the A and A+W treatments similar to those measured in the N+ treatment. This would indicate that the interplay between the photosynthetic and lipid biosynthesis pathways described for the diatom may not necessarily translate to *N*a*nnochloropsis*, although further analysis experiments in which the cellular redox state, the activity and expression of NR and the GLU/GLN ratio changes in this microalga growing in ammonium, nitrate or ammonium with W media or in NR knock-out mutants would be needed to confirm this hypothesis.

## Conclusions

In summary, in the present study, we aimed to investigate the combined effect of ammonium, a reduced source of nitrogen, and super-saturated light intensities in the accumulation of lipids in the microalgae *P. tricornutum* and *N. oceanica*. This comparative study allowed us to identify for the first time relevant differences in the physiology of these organisms regarding their acclimation ability to high light intensities and the degree of linkage between the photosynthesis and lipid biosynthesis pathways. While *P. tricornutum* growth was photoinhibited, lipid productivities under nitrogen starvation remained unchanged when compared to previous reports under sufficient light intensities, and a stimulation of lipid biosynthesis equivalent to that of the nitrogen starvation was observed when the organism was grown in ammonium. This lipid productivity was even higher when tungstate was substituted for molybdate in the ammonium treatment. Conversely, *N. oceanica* growth and physiology was not compromised by the high light intensities used in our study, and the use of ammonium instead of nitrogen starvation did not elicit an increase in the lipid productivities, but instead had a negative effect that was even more marked when tungstate was substituted for molybdate in the medium. These results point towards relevant differences between the two species in a way that suggests that NR plays a role in the linkage between the photosynthetic and lipid biosynthesis pathways, although further investigations with different light intensities and a detailed analysis of the oxidative stress and the NR activities and expression levels in the presence of ammonium, nitrate or ammonium with tungstate or in NR knock-out mutants would be necessary to obtain a better understanding of the response. Nevertheless, the results from this comparative study point at the direction of rationally optimising cultivation conditions for microalgal biofuel productions, and developing an appropriate understanding of the response by different species. This would enable the design of more effective industrial exploitations in which the lipid productivity of these organisms would be maximized by manipulating their culture conditions without the need for genetic engineering.

## Methods

### Experimental approach

*N. oceanica* (CCAP 849/10) and *P. tricornutum* (CCAP 1055/1) were obtained from the Culture Collection of algae and Protozoa (CCAP, Oban). The experiment consisted of two phases (Fig. 1), in which axenic cultures of *N. oceanica* and *P. tricornutum* were cultivated in flasks aerated with 0.22 µm-filtered moist air that were located inside a Sanyo Incubator (Panasonic model MLR-351) maintained at a constant temperature of 25 °C. In the first phase, cultures were grown to acclimatise them to the respective nitrogen source (nitrate or ammonium at 0.88 mM) and high light. To prepare the inoculum, the required volume of stock culture was harvested and subsequently washed with the respective medium (f/2 medium containing nitrate or ammonium as the nitrogen source), and re-suspended by gently pipetting in 500 mL of the same medium to an initial OD_595 nm_ of approx. 0.15. Cultures were then acclimatised to the experimental conditions by growing them in triplicate for 4 days and under continuous high light intensity (1000 µmol photons m^−2^ s^−1^) supplied by Osram Lumilux Cool White L36W/840 fluorescent lamps. Culture growth was followed by measuring OD_595 nm_ in a spectrophotometer (model ULTROSPEC 2100 PRO UV–Vis). At the end of the first phase, mean (± standard error) optical densities were 0.68 ± 0.03 and 0.57 ± 0.01 for *N. oceanica* and *P. tricornutum*, respectively.

In the second phase, cultures grown in the media with nitrate were harvested, and divided in two before washing and re-suspending them as described above: half of the centrifuged cells were washed and re-suspended in 250 mL of fresh f/2 medium with nitrate (at an initial concentration of 0.88 mM) as a source of nitrogen, and half of them were re-suspended in 250 mL of fresh f/2 medium without nitrogen. Cultures grown in the media with ammonium were also harvested and divided in two as described, half of the cells were re-suspended in 250 mL of fresh f/2 medium with ammonium as a source of nitrogen (at an initial concentration of 0.88 mM), and the other half were re-suspended in 250 mL of fresh f/2 medium with ammonium as a source of nitrogen and with the trace metal molybdenum substituted by tungsten (tungsten is a metal that occupies the place of the molybdenum in some enzymes such as N+ reductase) [57, 71]. After resuspension, cultures were grown in triplicate for 3 days under the same continuous high light intensity (1000 µmol photons m^−2^ s^−1^) and growth was followed by measuring OD_595 nm_. The maximum growth rate achieved was calculated in all the treatments as the maximum of the growth rate measured as ln(*C*_2_/*C*_1_)/(*t*_2 _− *t*_1_) between the monitored time points (*t*_1_ and *t*_2_).

### Determination of biochemical and elemental composition

At the end of the experiment, on day 3, 10 mL sample was taken from each replicate treatment to determine the final biochemical composition by centrifuging at 4500*g*, room temperature, for 5 min in an Eppendorf centrifuge model 5810. Pellets were washed with phosphate buffer (0.01 M) and kept at − 80 °C until subsequent analyses. Always working in the dark, chlorophyll *a* (chl a) samples were re-suspended in 2 mL of chilled 90% acetone and disrupted employing a bead-shaker (Genie) for 5 cycles of 2 min. Extracts were then centrifuged at 10,000*g* for 2 min in a Sorvall Legend Micro 17 Microcentrifuge and the absorbance of the supernatants at 630, 647 and 664 nm wavelengths determined in an ULTROSPEC 2100 PRO UV–Vis spectrophotometer to estimate the total chlorophyll *a* concentration using the following equations as reported in [72]: chlorophyll *a* (µg mL^−1^) = 11.8668 × abs (664 nm) − 1.858 × abs (647 nm) for *N. oceanica* and chlorophyll *a* (µg mL^−1^) = 11.4902 × abs (664 nm) − 4.504 × abs (630 nm) for *P. tricornutum*.

Total protein concentration was estimated by the Microbiuret method after doing an alkali extraction at 80 °C for 10 min in 3 mL of 0.5 N NaOH as reported in [73]. Total fatty acids concentration using palmitic acid as standard was estimated following the method detailed in [74]. Particulate organic carbon (POC) and nitrogen (PON) were determined in pre-weighted freeze dried samples in an Elemental Analyzer Flash 2000 using l-isoleucine as a standard. Finally, cell abundance was determined in samples fixed with Lugol in a Neubauer haemocytometer using a Zeiss Axiostar Plus Microscope.

### Photophysiological measurements

Chlorophyll *a* variable fluorescence was used to determine the changes in the photophysiology of *N. oceanica* and *P. tricornutum* under the different treatments. Samples concentrated to a final OD_595 nm_ of 3 were analysed in an Imaging PAM M-Series Chlorophyll Fluorescence System from Walz. The protocol employed to conduct the measurements was as follows: 600 µL aliquot samples were dark-adapted for 30 min before determining the maximum efficiency of PSII or maximum quantum yield (*F*_v_/*F*_m_) using a saturating light intensity of 100 µmol photons m^−2^ s^−1^ in the presence of a modulating light (ML) intensity of 15 µmol photons m^−2^ s^−1^. Subsequently, rapid light response curves (RLC) were conducted by increasing actinic light intensity every 30 s and measuring the light response of the quantum yield $\left( {{{F_{\text{v}} } \mathord{\left/ {\vphantom {{F_{\text{v}} } {F_{\text{m}} }}} \right. \kern-0pt} {F_{\text{m}} }}} \right)$ or Y(II), the photochemical quenching (qP), the non-photochemical quenching (NPQ) and the relative electron transport rates (ETR). ETR evolution was modelled by a Waiting-in-Line model as reported by [75] to estimate the maximum photosynthesis rate, *P*_max_ (measured as ETR), the optimum light intensity (*E*_opt_), and the maximum photosynthetic efficiency (*α*). After the RLC, samples were kept in the dark under the presence of ML for 5 min before measuring the qP again. The qP difference before and after the RLC is used as a proxy of photodamage to the photosystem II (PSII) [76]. Three technical replicates for each measurement were taken.

### Statistical analysis

The statistical differences between the treatments for the physiological parameters determined were analysed by performing an analysis of the variance (ANOVA) followed by the Bonferroni *t*-test using the SigmaPlot software version 13.
